# *Octopus insularis* as a new marine model for evolutionary developmental biology

**DOI:** 10.1242/bio.046086

**Published:** 2019-11-01

**Authors:** Ernesto Maldonado, Emma Rangel-Huerta, Roberto González-Gómez, Gabriel Fajardo-Alvarado, Piedad S. Morillo-Velarde

**Affiliations:** 1 EvoDevo Research Group, Unidad de Sistemas Arrecifales, Instituto de Ciencias del Mar y Limnología, Universidad Nacional Autónoma de México, Puerto Morelos, Quintana Roo, México 77580; 2 Doctorado en Ciencias Biomédicas, Universidad Nacional Autónoma de México, UNAM, México 77580; 3 Posgrado en Ecología y Pesquerías, Universidad Veracruzana, Boca del Río, Veracruz, México 94290; 4 Instituto de Ciencias Marinas y Pesquerías, Universidad Veracruzana, Boca del Río, Veracruz, México 94290; 5 CONACyT-Instituto de Ciencias Marinas y Pesquerías, Universidad Veracruzana, Boca del Río, Veracruz, México 94290

**Keywords:** Octopus embryo, EvoDevo, Culture

## Abstract

Octopuses are intriguing organisms that, together with squids and cuttlefishes, form the extant coleoid cephalopods. This group includes many species that can potentially be used as models in the fields of biomedicine, developmental biology, evolution, neuroscience and even for robotics research. The purpose of this work is to first present a simple method for maintaining *Octopus insularis* embryos under a laboratory setup. Second, we show that these embryos are suitable for detailed analyses of specific traits that appear during developmental stages, including the eyes, hearts, arms, suckers, chromatophores and Kölliker's organs. Similar complex traits between cephalopods and vertebrates such as the visual, cardiovascular, neural and pigmentation systems are generally considered to be a result of parallel evolution. We propose that *O. insularis* embryos could be used as a model for evolutionary developmental biology (or EvoDevo) research, where comparisons of the morphogenetic steps in the building of equivalent organs between cephalopods and known vertebrate model systems could shed light on evolutionary convergences and deep homologies.

## Abstract (precis)

**Summary:** Cephalopods are trending as new model organisms in development and EvoDevo. *Octopus insularis* embryos can be easily maintained in laboratory conditions and are amenable for live imaging.

## INTRODUCTION

Animal model systems have long been essential for the study of fundamental biological processes, including evolutionary processes, cellular physiology and pathogenesis. Specifically, the embryos of aquatic animals (e.g. zebrafish, sea urchin, sea anemone and octopus) present several experimental virtues, such as a small size, complete development outside the mother and low maintenance costs, all of which make them excellent models for studying developmental, genetic, physiological, biomedical and ecotoxicological processes (Golling et al., 2002; Rentzsch and Technau, 2016; Strähle et al., 2012). Furthermore, the optical clarity of aquatic animal eggs and embryos guarantees the observation of every developmental stage using microscopy and allows detailed experimental analysis from the first cell division through to the formation of embryonic germ layers and organogenesis (Boletzky et al., 2006). Finally, small embryos allow reasonable sample sizes to be tested together using multi-well plates to provide multiple experimental replicates at the same time, making them cost-effective animal models (Hill et al., 2005).

Coleoid cephalopods (octopus, squid and cuttlefish) exhibit the largest nervous systems found among invertebrates (Young, 1971) and a sophisticated visual system controlling body color changes for communication, camouflage and mimicry (Hanlon et al., 2011; Robin et al., 2014). Their skin is richly supplied with receptor cells that are responsive to tactile and chemical stimuli (Graziadei, 1971), and their prehensile arms are covered with powerful suckers that allow them to perform complex manipulative tasks (Tramacere et al., 2014). Moreover, they present strikingly sophisticated behaviors including complex problem solving, task-dependent conditional discrimination and observational learning (Fiorito et al., 1990; Fiorito and Scotto, 1992). Further studies of cephalopods may provide insights into aging processes, immunology, endocrinology and neurobiology, which are important areas of biomedical research, thus offering conclusions that could be extrapolated to other animal models, including humans. These characteristics, coupled with rapid growth rates and short life cycles, make them ideal candidates for research in many different disciplines (Vidal et al., 2014).

Cephalopods have also been used as models for studying developmental processes such as the formation of the cephalopod head complex (Shigeno et al., 2008) and the dynamics of octopus appendage formation and differentiation (Nödl et al., 2015) and have even been used to perform live observations of embryogenesis, which are particularly easy in these taxa given the relatively large size of their embryos (Boletzky et al., 2006). The study of cephalopod embryos could provide a comprehensive view of evolutionary developmental biology (EvoDevo) (Bassaglia et al., 2013; Bonnaud-Ponticelli, 2016; Navet et al., 2014). Cephalopods belong to the phylum Mollusca, a large group with approximately 80,000 extant species, representing 23% of all known marine organisms. This is an ancient group that originated in the Cambrian period approximately 541–485 million years ago for which 60,000–100,000 fossils have been described (Rosenberg, 2014; Taylor, 2007). For this reason, developmental comparisons between cephalopods and other model organisms such as the ecdysozoans *Drosophila melanogaster* and *Caenorhabditis elegans* and the vertebrate zebrafish (Antoshechkin and Sternberg, 2007; Guertin et al., 2010; Strähle et al., 2012) will provide insights for exploring deep homologies and evolutionary convergences.

The main goal of EvoDevo is to understand how developmental mechanisms influence evolution and how these mechanisms themselves have evolved (Breuker et al., 2006; Carroll, 2008). In this context, cephalopods may play an essential role as valuable models in EvoDevo research for several reasons. First, given their phylogenetic position (Lophotrochozoa, Mollusca), they provide a missing component for developmental studies. On the other hand, they present anatomically stunning convergent structures with vertebrates such as camera-type eyes and a central nervous system, thus offering abundant material to study forces directing the development and evolution of the metazoan nervous system (Bassaglia et al., 2013; Navet et al., 2014; Ogura et al., 2004).

However, successful studies in EvoDevo or developmental biology require the rearing of embryos in constant, favorable, healthy conditions, assuring all that physical parameters are maintained at constant levels (Lee et al., 2009c; Sykes et al., 2006). This is not an easy task since the egg surface acts as a good substrate for bacterial colonization, which decreases the oxygen concentration inside the eggs (Berg et al., 2013; Uriarte et al., 2011). For instance, bacterial growth can be triggered by a relatively low pH; therefore, *in vivo* methods require the maintenance of embryos in a very stable environment to allow proper development.

The aims of the present study were twofold: first, to present a new method for maintaining *O. insularis* embryos in culture from cleavage to hatching at a constant temperature using simple treatments based on sodium hypochlorite and filtered marine water; this method is useful for observing and recording live embryos under a binocular microscope. Second, to describe the potential of *O. insularis* embryos as a useful model for studying the development of specific structures such as the eyes, the three hearts, arms, suckers, chromatophores and Kölliker's organs of the octopus.

## RESULTS

### Embryo harvesting and treatment

**Fig. 1.**
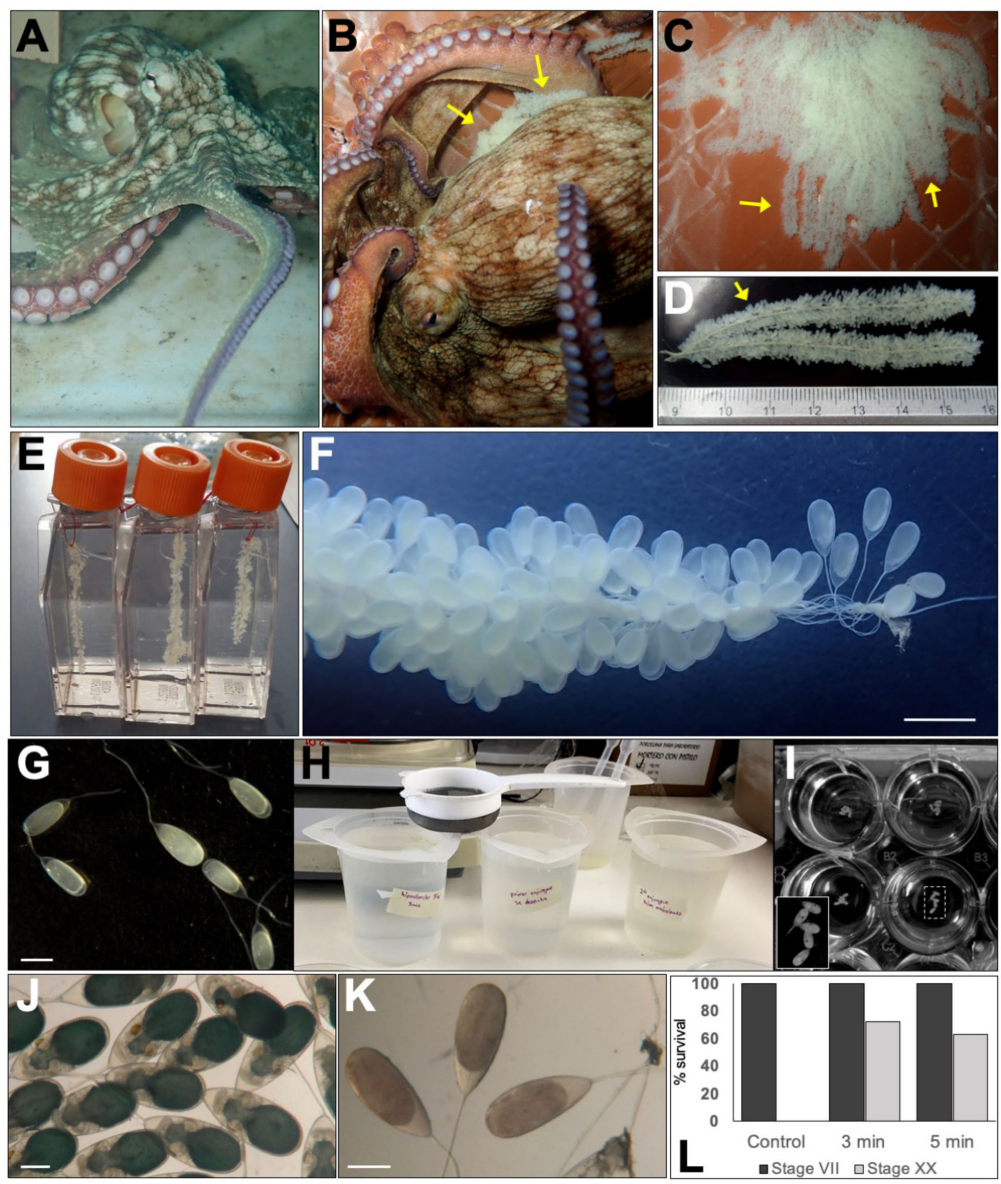
**Harvesting of embryos from *O. insularis* and bleaching treatment.** (A) An adult *O. insularis* specimen from the Veracruz Reef System. (B,C) *O. insularis* female with multiple egg strings (see arrows) hanging from the roof of the den. (D,E) Some strings were separated, placed in 200 ml containers and shipped to a different location. (F,G) Upon arrival, the strings were inspected, and each egg was separated from the string. (H) Groups of eggs were bleached by soaking them (using a strainer) in diluted NaOCl for 0 (control), 3 or 5 min, after which they were rinsed in FSW and bleached again. (I) The treated eggs were distributed in 12-well plates and placed in a 27°C incubator. (J) Bleached embryos developed rapidly after NaOCl treatment. (K) Non-bleached embryos did not develop and decayed within 2–3 days. (L) The survival rates from stages VII (dark bars) to XX (grey bars) were 0% for non-bleached embryos, 72.2% for embryos bleached for 3 min and 62.3% for embryos bleached for 5 min. Scale bars: (F) 3 mm, (G,J) 2 mm, (K) 1 mm.

Embryos were obtained from *O. insularis* captured from the Veracruz Reef System in Mexico (Fig. 1A). Octopus eggs incubated with parental care show high rates of survival, but this makes embryos difficult to obtain for developmental studies. Microorganism contamination of the chorion surface is often a problem for embryos under parent-free maintenance (Uriarte et al., 2011); for this reason, we decided to use a bleaching protocol that is employed for zebrafish embryos (Westerfield, 2000). *O. insularis* females lay their offspring in egg strings (Fig. 1B–D). For this analysis, three strings were gently separated from the mother (see Movie 1) and shipped overnight to a different facility. Upon arrival, the embryos were separated from the strings and treated in a diluted sodium hypochlorite (NaOCl) solution (see Material and Methods for details) (Fig. 1E–H), then maintained at a constant 27°C with daily filtered seawater (FSW) changes and were inspected every day to remove dead embryos.

Our aim was to achieve octopus development in a laboratory set-up. To do that, we tested our protocol in three different conditions: (I) non-bleaching, (II) bleaching for 3 min (twice) and (III) bleaching for 5 min (twice). The total number of embryos in our analysis was 998, among which 307 embryos were not bleached (control embryos), only rinsed with FSW. Another 356 embryos were bleached with diluted NaOCl for 3 min (twice), and 335 embryos were treated with diluted NaOCl for 5 min (twice). Within a few days, all non-bleached control embryos died, while 257 and 211 of the embryos treated for 3 and 5 min, respectively, survived from developmental stage VII–XX (Fig. 1I–L). The fact that none of the non-bleached embryos survived whereas an average of 67.6% of the bleached embryos did (Fig. 1L) suggests that bleaching is essential for *O. insularis* embryos when they are maintained without maternal care in an incubator under laboratory conditions. We did not observe any significant difference between the development of the embryos bleached for 3 or 5 min.

### Eye development in *O. insularis*

**Fig. 2.**
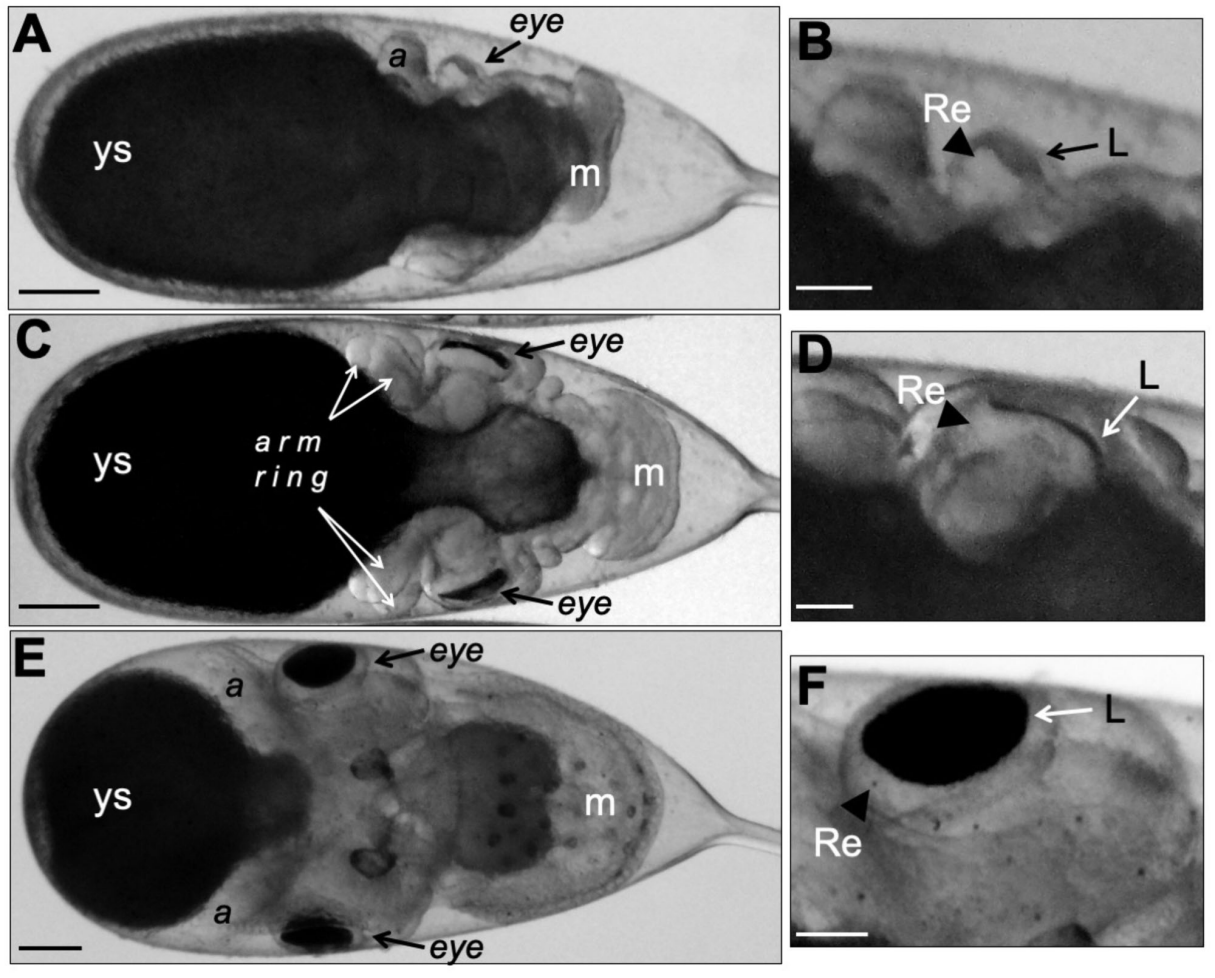
**Eye development in *O. insularis* embryos.** (A) *O. insularis* embryo at stage X; one of the developing eyes can be observed between one arm and the mantle. (B) At higher magnification, in the same embryo's eye, the retina (arrowhead) and the lenses (arrow) can be distinguished. (C) Dorsal view of a stage XII *O. insularis* embryo, in which the eye stalks protrude from the head. The arm ring is clearly visible. (D) The same embryo at a higher magnification. Arrowhead and arrow point to the retina and the lenses, respectively. (E) At stage XVI, the eyes have grown, becoming prominent structures of the embryo. Eye movements can be clearly observed. (F) At higher magnification, the lens (arrow) has a distinctive round shape located above the retina (arrowhead). A, arm; ys, yolk sac; m, mantle; Re, retina; L, lens. Scale bars: (A,C,E) 250 µm, (B,D,F) 100 µm.

Comparisons of eye function and morphology between cephalopods and vertebrates are of interest because it is generally accepted that the eyes of the species in these two groups evolved separately. Therefore, studies on eye development in *O. insularis* embryos could be significant. The early stages of eye development in *O. insularis* were observed at developmental stage X (Fig. 2A,B), after which there was considerable growth; by stage XII, the eye stalks protruded from the head, and eye pigmentation became obvious. Both eyes are located adjacent to the arm ring and above the structures forming the gills and the mantle (Fig. 2C,D). At stage XV, the development of the eyes of *O. insularis* was considerably advanced; the eyes even showed movement, which indicates the development of extraocular muscles, while the yolk sac had decreased in size (Fig. 2E,F).

### Development of branchial and systemic hearts in *O. insularis*

**Fig. 3.**
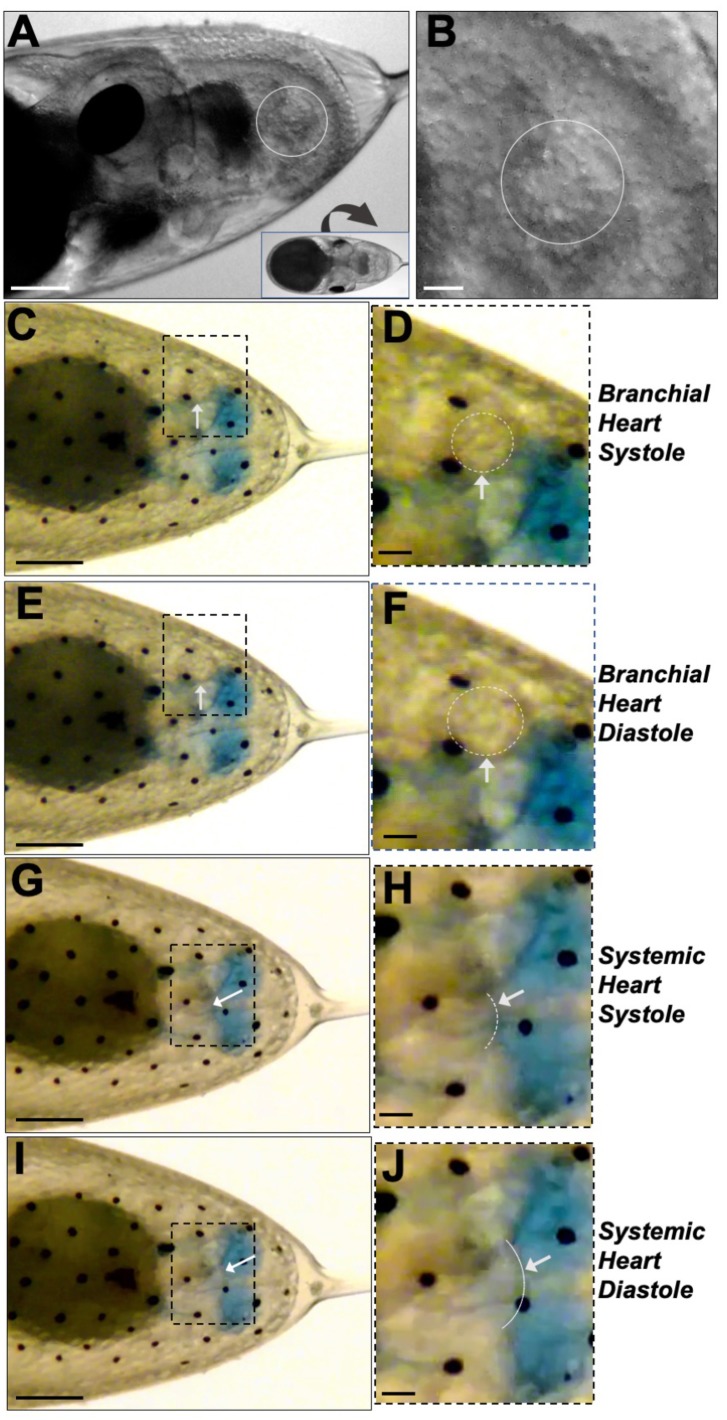
**Heart development in *O. insularis* embryos.** (A,B) Around developmental stage XV, we detected the initial heartbeats from *O. insularis* embryos. Octopuses develop two branchial hearts and one systemic heart. (A) The embryo was rotated (see inset) to obtain a dorso-lateral view to analyze one of the branchial hearts (white circle). (B) A magnified view shows concentric cells forming a branchial heart (white circle). (C,E,G,I) Ventral side views of a stage XVIII *O. insularis* embryo. Boxed areas are selected regions magnified in D,F,H and J. (C,D) A contracted branchial heart (systole) and (E,F) the relaxed heart (diastole). (G,H) The systemic heart in systole and (I,J) in diastole. White arrows show the area where the hearts are located. White circles show one of the branchial hearts. White dashed semicircular lines show the systemic heart. The blue-blotched area corresponds to hemocyanin-enriched blood. Scale bars: (A,C,E,G,I) 250 µm, (B,D,F,H,J) 50 µm.

*O. insularis* is a unique model in which to study heart development. Octopuses, squids and cuttlefishes possess three hearts: two branchial hearts and one systemic heart. The branchial hearts help oxygenate blood in the gills and are connected to the systemic heart, which pumps blood to the whole body. We detected the first sign of a heartbeat at developmental stage XV, when we registered consistent heartbeats in two lateral regions on the posterior side of the embryo inside the mantle (Fig. 3 and Movie 2). Because of their location, we believe these to be the branchial hearts. In Movie 2, the branchial heart at the top can be observed to beat approximately every 2 s (6 beats in 11 s), while the one at the bottom beats irregularly (4 beats in 11 s). It seems that the two hearts do not beat in coordination at this point. We also observed some contractile movements at a region located between the two branchial hearts, however, at this point, we could not discern whether these movements corresponded to the systemic heart. One of the branchial hearts showed cells arranged in a concentric circle that moved with every heartbeat (Fig. 3A,B and Movie 2).

At developmental stage XVIII, we could differentiate the beating of the branchial hearts from the systemic heart only because we noticed that during development, the systemic heart suddenly stopped beating, only to be restarted a few seconds or minutes later. This phenomenon did not occur in the branchial hearts. Movie 3 shows a ventral view and starts with the beating of only the branchial hearts; a few seconds later, the systemic heart begins to beat. At this developmental stage, the three hearts showed a similar heart rate. The heart rate of one of the branchial hearts was one beat every 1.02 s (0.069 SD) and that of the other branchial heart was one beat every 0.91 s (0.055 SD). The systemic heart rate was one beat every 0.93 s (0.065 SD) (Movie 3). The systole (contraction) and diastole (relaxation) phases of heart function could be distinguished for both the branchial hearts (Fig. 3C–F) and the systemic heart (Fig. 3G–J).

Systolic movements seemed to alternate between the systemic and branchial hearts. To confirm this, we recorded the movements of both types of hearts from a lateral view. In Movie 4, it is shown how a single systole-diastole cycle of a branchial heart is followed by a systole-diastole cycle movement of the systemic heart. The branchial heart is in a more ventral position than the systemic heart. The systolic and diastolic movements in *O. insularis* embryo's hearts suggest that these may be pumping blood through a closed circulatory system at this developmental stage; however, we could not detect any fluid movement around the embryo body. Cephalopods are blue-blooded because the copper-rich hemocyanin protein transports oxygen in these species. In stage XVIII embryos, we observed a blue blotchy region adjacent to the systemic heart (Fig. 3C–J), which could be related to hemocyanin oxygenation.

### Arms and sucker development in *O. insularis*

**Fig. 4.**
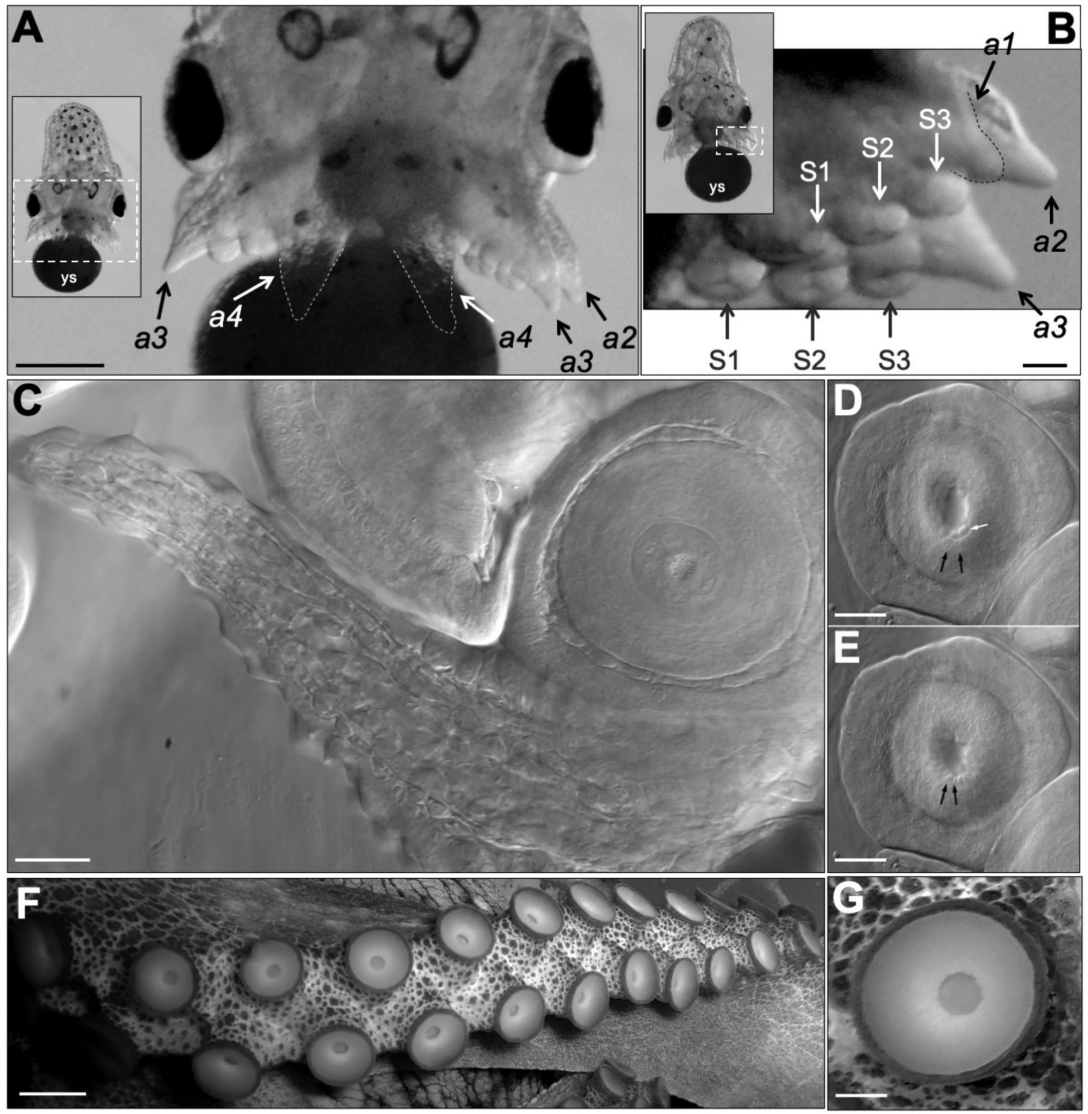
**Arm and sucker development in *O. insularis* embryos.** (A) Ventral view of the arms of a dechorionated stage XVIII *O. insularis* embryo. The inset provides a panoramic view of this embryo. The arms are numbered from dorsal to ventral, and the most ventral arms are indicated with dotted lines. (B) Magnified view of the arms and suckers from a dorsal view (see inset). Arm 1 (a1) is out of focus; therefore, the labeled suckers are from arms 2 (a2) (white labels, S1–3) and 3 (a3) (black labels, S1–3). Each arm exhibits three suckers until hatching, which are numbered from proximal to distal. (C) DIC microscopy image of one arm and two suckers. (D–E) The suction motion of one sucker was detected when centrally located cells moved towards the center. The cell closest to the center (white arrow) disappeared from focus, and two contiguous cells (black arrows) also moved inwards. (F) One arm and suckers from an adult *O. insularis* specimen. (G) Magnification of one sucker from this adult. Scale bars: (A) 250 µm, (B) 50 µm, (C,D,E) 25 µm, (F) 3 cm, (G) 250 µm.

In *O. insularis*, the arm ring formed very early in development at approximately stage VII–VIII, as in *Octopus* *vulgaris* (Naef, 1928). We show the arm ring in *O. insularis* at stage XII, seen from a dorsal view of the embryo (Fig. 2C). The embryo's arms were difficult to study because they grew surrounding the opaque yolk sac. We removed one octopus embryo from its chorion at stage XVIII using fine-tipped forceps, after which the arms and suckers became easier to observe in both ventral and dorsal views (Fig. 4A,B). The arms were numbered in the dorsal to ventral direction from a1 to a4 on each side, as in Naef (Naef, 1928). From the ventral and dorsal views, we could observe arms a2 to a4, which were 320–360 µm in length. The embryo had a total of 24 suckers (three per arm), which were numbered proximal to distal and had a diameter of 90–120 µm at this developmental stage (Fig. 4A,B). As in other octopus species, no additional suckers were formed prior to hatching.

For more detailed observation of the arms and suckers, we placed a dechorionated stage XVIII *O. insularis* embryo in an agarose pad chamber filled with FSW and observed it using differential interference (DIC) microscopy (Fig. 4C and Movie 5). At stage XVIII, the arm consists of different cell types, including several layers of myocytes forming longitudinal muscle fibers that can be observed along the arm in Fig. 4C and Movie 5. The suckers are required for many arm functions; these chemo-tactile structures are composed of radial, meridional and circular muscular fibers, including a sphincter muscle (Nödl et al., 2015). We found that sucker suction motion could be observed in detail using DIC microscopy. At this developmental stage, we observed that the interior part of the sucker exhibits an internal cavity known as the acetabulum and one external cup-shaped structure known as the infundibulum. Additionally, we observed that the orifice connecting the infundibulum with the acetabulum was encircled by only 11–14 cells. When the sphincter muscle contracted, the cells encircling the orifice shifted towards the center, after which they returned to their position as the sphincter muscle relaxed. This sucker suction motion was quick, lasting only for 0.5–0.8 s (Fig. 4D,E and Movie 5). For comparison, we show the *O. insularis* adult suckers, which form two rows on the oral side of the arm and vary in size depending on their position along the arm (Fig. 4F–G).

### Chromatophores and Kölliker's organs

**Fig. 5.**
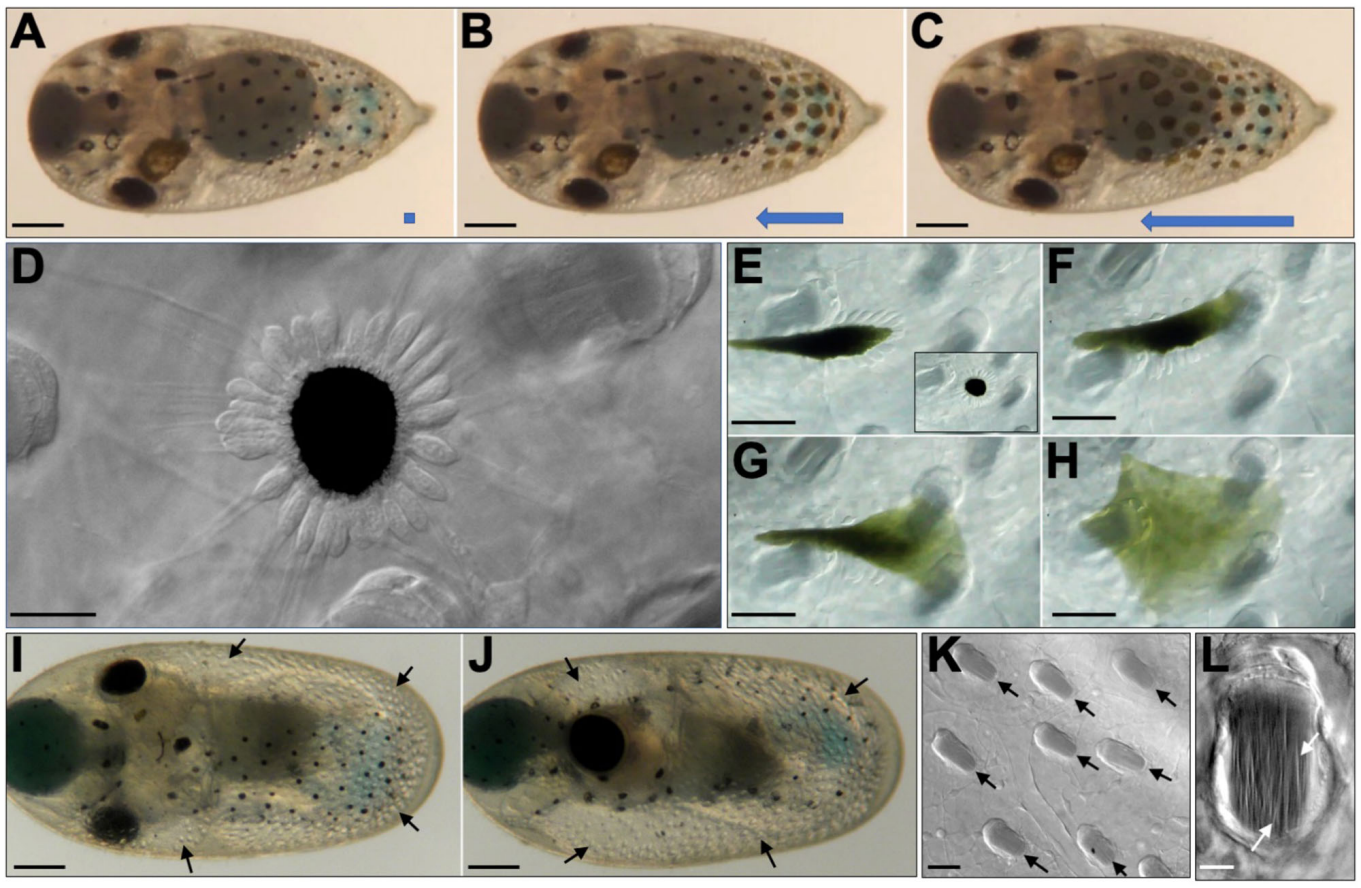
**Chromatophores and Kölliker's organs during development in *O. insularis* embryos.** (A–C) A ventral view of a Naef's stage XVIII embryo during a wave of chromatophore expansion–retraction. (A) The expansion–retraction wave was not yet initiated. (B) Only the most posterior chromatophores were expanding (follow the blue arrow). (C) The medial body chromatophores were expanding, while the more posterior ones were already retracting. (D) DIC microscopy image of a retracted chromatophore surrounded by 27–28 muscle fibers. (E–H) Sequential images of chromatophore expansion. (E, inset) A chromatophore just before expansion. (E) Muscular fibers pulled the chromatophore to the left side. (F) Fibers at the upper right corner pulled the pigmented cell. (G) Thereafter, the chromatophore was extended to the right by muscular fibers on that side. (H) At the end of the time sequence, the chromatophore was fully expanded. (I,J) Dorsal and lateral views, respectively, of a Naef's stage XX *O. insularis* embryo showing Kölliker's organs at the epidermis (black arrows). This embryo has already undergone the second inversion (see text). (K) A set of Kölliker's organs observed using DIC microscopy, which were oriented in the same direction. The arrows have the same orientation as this set of Kölliker's organs. (L) Multiple rodlets (white arrows) inside a single epidermal Kölliker's organ that were observed by DIC illumination. Scale bars: (A–C) 300 µm, (D) 20 µm, (E–H) 50 µm, (I,J) 250 µm, (K) 50 µm and (L) 10 µm.

Except for eye coloration, no pigmentation was observed during *O. insularis* early developmental stages. Skin chromatophores became visible at stage XV of development. The mimetic ability of cephalopods to blend with their surroundings is remarkable and is achieved in part by the ability of chromatophores to expand and retract. Similar to other cephalopods, *O. insularis* can adjust its body color to blend with its surroundings (compare Fig. 1A and B). We found that skin chromatophores from the ventral side of *O. insularis* stage XVIII embryos spontaneously expanded and retracted in a wave traveling in a posterior to anterior direction (Fig. 5A–C and Movie 6). We also noted that not all chromatophores expanded to the same extent. Some chromatophores were selected and measured before and after expansion. Some chromatophores located in central regions on the ventral side of the body expanded by an average of 16.6 times (min. 1.83 and max. 39.7 times), while chromatophores in the most anterior regions on the ventral side expanded by an average of only 2.6 times (min. 1.08, max. 3.63 times) (Fig. S1).

It is known that chromatophore expansion and retraction in cephalopods are achieved by the contraction of radial muscle fibers that surround the cells and are controlled by motor neurons (Messenger, 2001). For the purpose of observing chromatophore expansion in detail, we placed a dechorionated stage XX *O. insularis* embryo embedded in 1% agarose inside a chamber filled with FSW (see Material and Methods section). The chamber was placed on an upright microscope, and images were taken using a 40× objective under DIC illumination. We counted 27–34 muscle fibers (Fig. 5D) surrounding the chromatophores, and we believe that this number depends on chromatophore size during development. To induce chromatophore expansion under these conditions, we exposed the embryos to a fluorescence light with a rhodamine filter for a few seconds, and as a result, chromatophore expansion was induced (Fig. 5E–H and Movie 7). However, under this setup, the chromatophores expanded at a much slower speed than in non-mounted embryos under the stereoscopic microscope. Chromatophore expansion was uneven because fibers pulled this pigment cell from different directions but not at the same time. This behavior was also observed in the chromatophores of embryos inside their chorions under a stereoscopic microscope (data not shown).

One prominent feature of *O. insularis* skin is that it is covered by small protruding blobs or bumps, giving the embryo's epidermal surface a punctate appearance when illuminated from certain directions (Fig. 5I–J). These protruding structures are known as Kölliker's organs and have been described in other octopus species. These transient epidermal projections have the appearance of bristles composed of a tuft of cannular rodlets attached to a single epidermal follicle when expanded (Brocco et al., 1974). Their function is not clear, but it has been suggested to be related to the hatching process (Boletzky, 1966). In *O. insularis*, Kölliker's organs are formed in late embryogenesis and remain retracted during development. All of these structures were oriented in the same direction with their longest axis towards the anterior end (Fig. 5I–K). Richard A. Cloney and collaborators showed that the Kölliker's organs of *Octopus spp.* and *Eledone moschata* show a tuft of approximately 1500 rodlets when everted (Brocco et al., 1974). We detected these rodlets by DIC microscopy inside of some of the Kölliker's organs of *O. insularis* embryos (Fig. 5L).

## DISCUSSION

To promote developmental studies in the octopus, a simple protocol is required to achieve the survival of a high number of embryos under a simple laboratory setup, even when embryos are shipped from a different facility. In this work, we show that *O. insularis* embryos develop with a good survival rate after treatment in a diluted solution of NaOCl when they are maintained at 27°C with daily water changes. While octopus eggs maintained with parental care are more likely to survive, it has been reported that cephalopod embryos reared separately from the mother but maintained in aquarium tanks under a continuous flux of highly aerated water can survive. This approach has been tested in cuttlefishes (Nabhitabhata, 2014), squids (Nabhitabhata and Ikeda, 2014; Vidal and von Boletzky, 2014) and octopuses (Rosas et al., 2014).

Our bleaching protocol (tested with close to 1000 embryos) greatly increased embryo survival compared to non-bleached embryos when the embryos were maintained inside an incubator in petri dishes or multi-well plates. This could have practical applications for the continuous monitoring of octopus development under a microscope, for example. In a similar protocol, bobtail squid embryos (*Euprymna scolopes*) not subjected to any pretreatment with antiseptic agents have been maintained in a shaker incubator (40 rpm at 24°C), where continuous agitation improves aeration (Lee et al., 2009a,b). For *Octopus maya*, the cap of the mother nest was removed after spawning and placed in an incubator with sea water recirculation under controlled conditions, and this system was as effective as when the mother nurses the embryos (Rosas et al., 2014). The use of NaOCl as a pretreatment for octopus embryos is a potential option when water recirculation tanks are not desired or not available.

However, the use of NaOCl may not be appropriate for all experimental conditions, particularly if the oxidative stress response is to be studied during octopus development, since NaOCl is known to produce reactive oxygen species (ROS) in different animal models (Amorim et al., 2017; Salinas et al., 2006). Notably, the fact that *O. insularis* embryos can be maintained at high numbers and develop correctly in multi-well plates at a density of one embryo/0.1 ml suggests that *O. insularis* embryos may also be suitable for genetic, chemical or toxicological screening. The second aim of this work was to show that once an adequate number of embryos is obtained in a laboratory setup and without maternal care, many aspects of *O. insularis* development can be studied in detail. In particular, eye and heart development, arm and sucker patterning, Kölliker's organ formation and the mimetic system of chromatophore expansion and retraction can be investigated.

We show that eye formation can be easily monitored in *O. insularis* embryos by following eye development and growth from early stage X to late stage XX. Eye movement was detected from embryonic stage XVI onward, suggesting that an extraocular muscle system controlling eye position already exists at this developmental stage. *O.* *insularis* embryos seem to be a tractable system for performing studies addressing how visual function is acquired during development. In the squid *Doryteuthis pealeii,* a detailed fate map during eye development was produced (Koenig et al., 2016). These authors found that the Pax6, Pax2, Six3, Eya, Hes, Prospero and Notch genes play a crucial role in squid eye morphogenesis. Some of these transcription factors (particularly Pax6) are required for eye development by other bilateral animals. It will be of interest to determine whether these genetic pathways are conserved in *O. insularis* during development. The evolutionary origin of eyes remains controversial since, based on morphology and physiology, the eyes of different groups of animals do not seem to have a common origin (Salvini-Plawen and Ernest, 1977), while genetic evidence shows that eye formation in different metazoan lineages is controlled by the same set of transcription factors (Gehring, 2014).

We were able to follow the formation of the three hearts of *O. insularis*. Based on our observations, we suggest that function of the branchial hearts may be initiated before that of the systemic heart. Branchial hearts start to beat around stage XV; however, we did not detect regular heartbeats of the systemic heart until stage XVIII; therefore, the branchial hearts may be the source of the cardiac rhythm for the systemic heart. Accordingly, it has been suggested that in *O. vulgaris* adults, cardiac ganglions from the branchial hearts are likely sites of pacemaker function (Wells, 1980). During developmental stage XV, the *O. insularis* branchial hearts beat at a slower pace of approximately one beat every 2 s, and they do not beat at the same time, whereas in stage XVIII, these hearts beat faster (one beat per s) in a regular and synchronic manner (compare Movie 2 to 3). Thus, some maturation or pacemaker tuning of the branchial hearts may take place between stages XV and XVIII. Heartbeat frequency is affected by temperature and oxygen levels, and not all cephalopods exhibit the same heart rate; for example, the *Nautilus* heartbeat is much slower than those of octopuses, and both taxa exhibit slower heartbeats than squids (Wells, 1992).

We observed that the *O. insularis* embryo systemic heart suddenly stopped beating and later restarted either by itself or after a whole-body contraction. In one reported experiment, one *O. vulgaris* male missed a systemic heartbeat when a female was introduced to the same tank (Wells, 1979). In other species such as *Enteroctopus dofleini*, the systemic heart can stop for up to 1 h without any apparent ill effects (Wells, 1979). There is extensive knowledge about the genetic pathways and cellular differentiation events related to heart development in vertebrates (Perl and Waxman, 2019; Zhang, 2019). As shown in this work, heart function during developmental stages could be easily visualized in *O. insularis* embryos; therefore, this octopus may be an excellent model for understanding how cephalopods and vertebrates reached similar solutions to make oxygen available to the cells in their bodies.

In *O. insularis,* the arm suckers first appeared around stage XIII, and by stage XIX, they were fully formed. Arm motility and sucker suction movements were easily observed in dechorionated embryos using DIC microscopy. Similar observations have been reported in other octopuses such as *O. vulgaris* and *Euprymna scolopes* (Nödl et al., 2015, 2016). The dexterity of the flexible arms in coleoid cephalopods has no equivalent in other bilateral groups. Many animals such as birds, primates and rodents can also grab things but not with the grip strength and precision of the octopus's eight arms covered by suction cups or suckers.

Octopus arm suckers possess a complex structure consisting of two parts: the infundibulum (exposed disk-like portion) and the acetabulum (hollow internal chamber) (Kier and Smith, 2002). Suckers maintain a grip on objects for long periods of time without muscular energy consumption due to the negative pressure produced when the acetabular protuberance seals the orifice between both compartments (Tramacere et al., 2013). By observing suction motions in suckers from *O. insularis* embryos, we detected that by developmental stage XX, only a few cells surround the orifice connecting the infundibulum and the acetabulum. When the sucker initiates suction, these cells are drawn to the interior part of the orifice (Movie 5). *O. insularis* embryos could be used to investigate how early in development suction function appears.

Suckers harbor sensory cells, and each adult arm may contain 30 million neural receptors, including mechanoreceptors, proprioceptors and chemoreceptors (Graziadei, 1971). Transcriptomics experiments in *Octopus* *bimaculoides* showed that suckers express a group of atypical acetylcholine receptor-like genes and a set of paralogous genes from the G-protein coupled receptor (GPCR) family (Albertin et al., 2015). After hatching, *O. insularis* larvae immediately need to obtain nutrients. It could be tested whether these particular receptors are also expressed during sucker development in *O. insularis* embryos under our experimental conditions. Furthermore, because *O. insularis* embryos develop well in a multiplex format, it may be possible to carry out genetic screening to identify chemoreceptor genes that are specific for certain flavors or chemical substances. Arm suckers have an additional function: males use them for display in courtship or deimatic behavior against potential predators (Packard, 1961).

The mating display in cephalopods also includes changes in coloration; for example, cuttlefish males display zebra-striped patterns on their arms (Hanlon and Messenger, 1988). Our group has shown that *O. insularis* males show a bicolor pattern for courtship (Fajardo-Alvarado et al., 2018). Coloration changes in coleoid cephalopods are produced by neural control over muscles that expand or retract skin chromatophores. Two dynamic patterns, referred to as passing clouds and wandering clouds (moving dark stripes on the body), have been observed in squids, cuttlefishes and octopuses (Laan et al., 2014; Packard and Sanders, 1971). While passing clouds are controlled by circuits upstream of chromatophore motoneurons (Laan et al., 2014), wandering clouds are produced by the electrical coupling of chromatophore muscles independent of neural inputs (Messenger, 2001).

We observed an embryo version of a traveling wave of chromatophore expansion and retraction in *O. insularis* embryos (stage XVIII) moving from the mantle to the arm ring (Movie 6). It is not uncommon to observe dynamic coloration changes when cephalopod embryos are developing. *Sepia officinalis* larvae show intricate coloration patterns when they hatch (Hanlon and Messenger, 1988). We offer two alternative scenarios regarding how chromatophore expansion and retraction waves are regulated in *O. insularis* embryos. Their regulation could be only myogenic or wandering-cloud-like if control neurons have not yet developed, or it could be passing-cloud-like if it is generated by central pacemakers that are active in developmental stages.

The speed with which cephalopods mimic their background is unmatched in bilateral animals; it is achieved by rapid shape changes in chromatophores. Each chromatophore consists of a single cell full of pigment that, at rest, is highly folded and elastic. It is attached to several muscle cells that achieve chromatophore expansion by contracting (Messenger, 2001). Chromatophore neural control arises from the optical and chromatophore lobes and implies a complex network, since every radial muscle is innervated by approximately six excitatory motoneurons and receives up to 100 synapses (Dubas et al., 1986; Florey, 1969; Messenger, 2001). Octopuses exhibit different numbers and types of chromatophores, including black, red, yellow, orange and brown chromatophores. Black chromatophores are under the control of L-glutamate, while the others (except for brown chromatophores) are regulated by dopamine (Messenger, 2001). Not all of these chromatophore types appear at the same time during development (Messenger, 2001).

In this work, we only observed black chromatophores during the development of *O. insularis* embryos; other types of chromatophores may not be easily identifiable at these stages or may appear later. Most of the current knowledge about chromatophore function comes from experiments performed in adults. The *O.* *insularis* embryos maintained under our experimental conditions proved to be amenable for live imaging, which allowed us to induce and observe chromatophore expansion (Movie 7). Muscular cells attached to chromatophores produce graded contractions; as expected, we observed intermediate chromatophore expansion states in *O. insularis* embryos. The rich, diverse coloration patterns observed in *O. insularis* adults are the outcome of dynamic and coordinated networks among different types of cells, not only chromatophores but also iridocytes, leucocytes and reflector cells. *O.* *insularis* embryos may be a suitable model for understanding how this fascinating network originates during development.

## MATERIAL AND METHODS

### Octopus culture and egg strings collection

Several *O. insularis* individuals were identified in the shallow waters of the Veracruz Reef System (southwestern Gulf of Mexico) based on coloration patterns outlined in González-Gómez et al. (2018) and caught with the help of artisanal fishermen. The reproductive season for this particular octopus species is from February to May. Once caught, the specimens were transported to the laboratory in plastic coolers with aerated seawater. After reaching the laboratory, the animals were transferred to 700 l flow-through tanks with mechanical and biological filter systems and maintained at a 1:1 male:female ratio at ambient temperature (27–30°C) with salinity between 33–35 ppm and pH 8.0–8.3. The tanks contained ‘casitas’-like ceramic shelters and an external 0.5-inch Raschel net to prevent the animals from escaping and to stimulate gonadal maturation (Zuñiga et al., 1995). The animals were fed frozen crabs (*Callinectes sapidus*), snails (*Strombus pugilis*) and discarded fish on alternate days 6 days a week. Once mating was observed, the males were removed from the tanks. Seven days after spawning, three egg strings were obtained (Movie 1). The strings were shipped overnight inside culture bottles filled with seawater to the Instituto de Ciencias del Mar y Limnología, UNAM, Puerto Morelos, Mexico, for treatment and observation.

### Sodium hypochlorite treatment and embryo culture

Embryos arrived at approximately stage VII of development and upon arrival, they were immersed in a glass beaker and rinsed with FSW. Then, the embryos were individually detached from the egg strings using fine scissors, and a total of 998 embryos were obtained. The embryos were next split into three sets, which were treated under the same conditions except that the time of exposure to NaOCl was 0 (control) and 3 or 5 min. The diluted NaOCl solution was prepared by adding 180 µl of 10–13% NaOCl (Sigma-Aldrich, 425044) to 500 ml of FSW. For treatment, the octopus embryos were placed in a plastic strainer and submersed in diluted NaOCl for 0, 3 or 5 min. Thereafter, the embryos were rinsed in a beaker with 500 ml of FSW for 1 min and immersed again in the diluted NaOCl solution for the same time as in the first treatment. The embryos were subsequently rinsed in two different beakers with 500 ml of FSW in each case for 1 min in the first container and 10 min in the second one. This procedure was based on a treatment that is commonly used to bleach zebrafish embryos (Westerfield, 2000). After the diluted NaOCl treatment, the octopus embryos were separated in petri dishes (60 embryos per dish), 12-well plates (10 embryos per well) or 24-well plates (6 embryos per well) containing FSW and maintained in an incubator (Boekel-Scientific, 133000) at 27°C. Daily FSW changes were carried out along with the inspection and removal of dead embryos.

### Microscopy

Embryos were placed in FSW on concave slides for photography and video recording. Images and videos were recorded with a Sony camera (DSC-H20) attached, with an MM99 camera adaptor (Martin Microscopes), to a stereoscopic microscope (Nikon, SMZ645) with a diascopic illumination base. DIC microscopy was carried out using an AxioImager microscope equipped with an AxioCam MRc camera and ZEN image capture software (Zeiss). All images were processed with Adobe Photoshop CC and supplementary movies were edited with the Wondershare video converter and iMovie 10.1. For live DIC microscopy, after the removal of the chorion, embryos were embedded in 1.5% low-melt agarose (Sigma-Aldrich, A9414) prepared with FSW and placed in a chamber filled with FSW. A number 0 coverslip (Electron Microscopy Sciences) was gently placed over the immobilized embryo. The embryo must be in direct contact with the coverslip for proper imaging.

### Designation of body axes, heartbeat counts and chromatophore area measurements

The developmental staging of *O. insularis* embryos and the designation of their dorso-ventral and antero-posterior regions were based on a detailed description of *O.* *vulgaris* development by Adolf Naef (Naef, 1928). We chose these designations, although the allocation of body axes in cephalopods is controversial (Shigeno and Von Boletzky, 2010).

The heartbeat rates of the branchial hearts of embryos at stage XV (Movie 2) and stage XVIII (Movie 3) were calculated by counting the number of heartbeats over 10 or 15 s, respectively. The corresponding standard deviation was calculated using Microsoft Excel. The chromatophore area before and after expansion was calculated using the Fiji module ‘Particle Analyzer’ (Schindelin et al., 2012) (Fig. S1). For this analysis, chromatophores were hand-picked in mid-body regions and anterior regions (a similar number in each case) from still images in Movie 6.

## Supplementary Material

Supplementary information
