# Supplementary material for: Octopus insularis as a new marine model for evolutionary developmental biology
Source: Biol Open. 2019 Nov 1;8(11):bio046086. doi: 10.1242/bio.046086 (PMC6899024; doi:10.1242/bio.046086)
Supplement: Supplementary information [file biolopen-8-046086-s1.pdf]

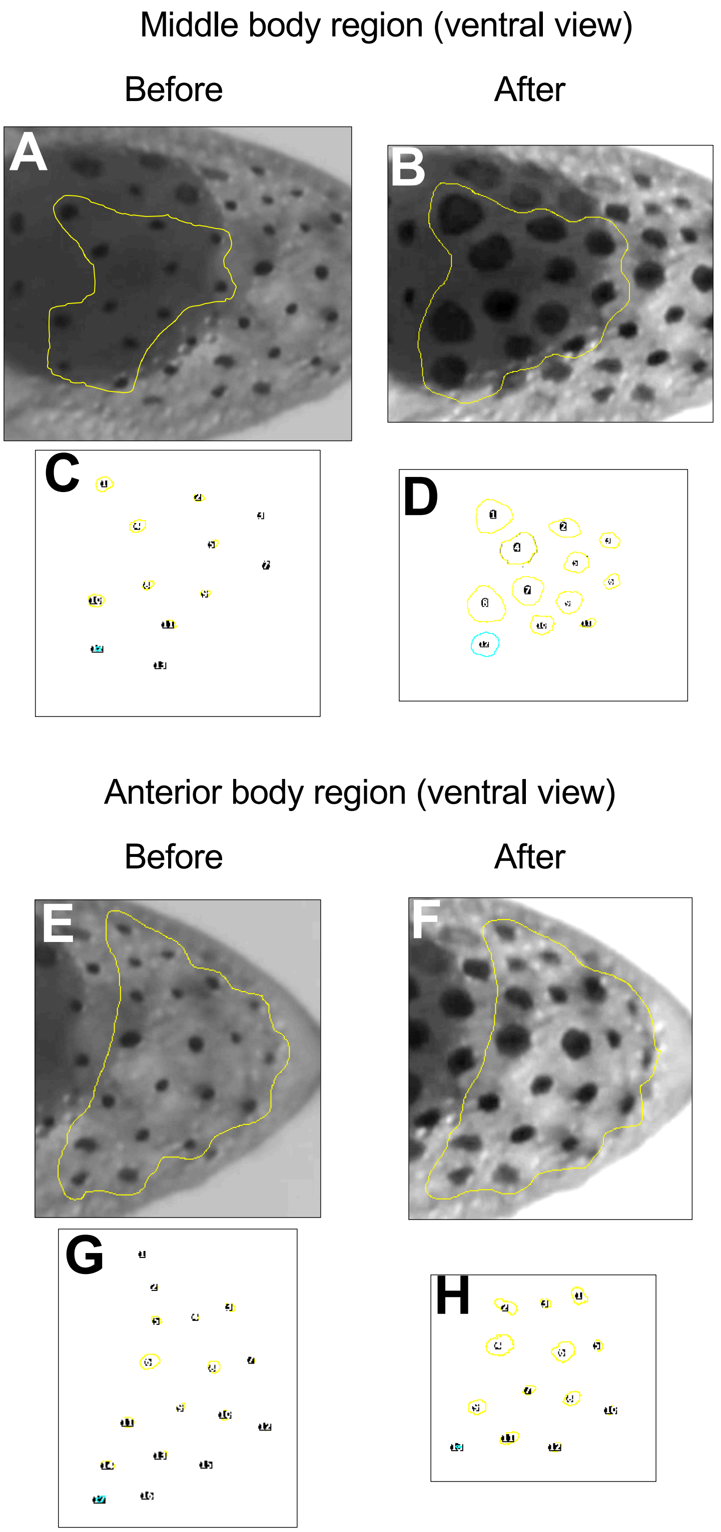

Biology Open • Supplementary information

Figure S1. Analysis of chromatophore expansion. (A, B, E and F) Still images were taken from Supplementary Movie 6 to determine the extent of chromatophore expansion in different regions of a stage XVIII embryo. (A – D) Chromatophores in the mid-body section, before and after expansion; some were selected for area measurement (C – D). Chromatophores from the anterior region of the same embryo; some were chosen (G – H) to measure their size before and after expansion. Fiji software was used for this analysis.

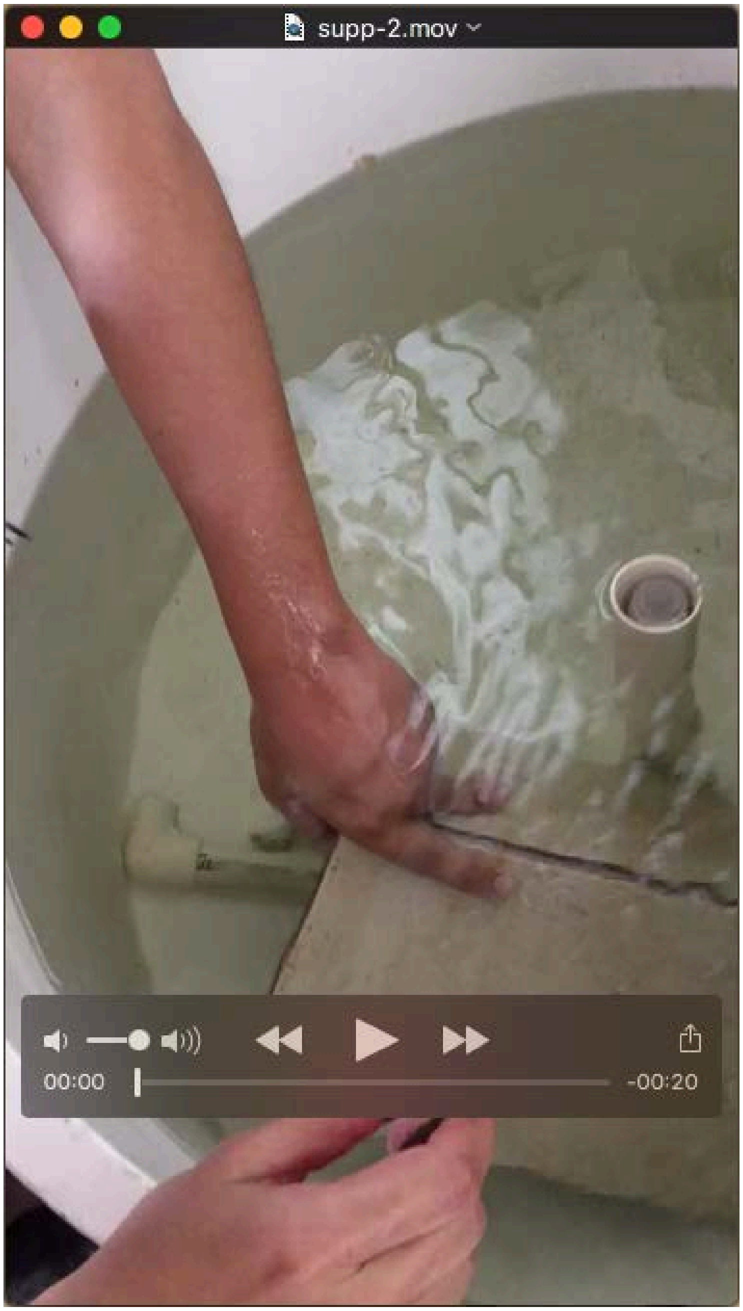

Movie 1. Harvesting of egg strings from one *O. insularis* female. While one person lifts the shelter, another person holds and cuts the required egg strings using scissors and then places the string in a petri dish prefilled with FSW. This must be done swiftly to avoid stressing out the octopus female.

Biology Open • Supplementary information

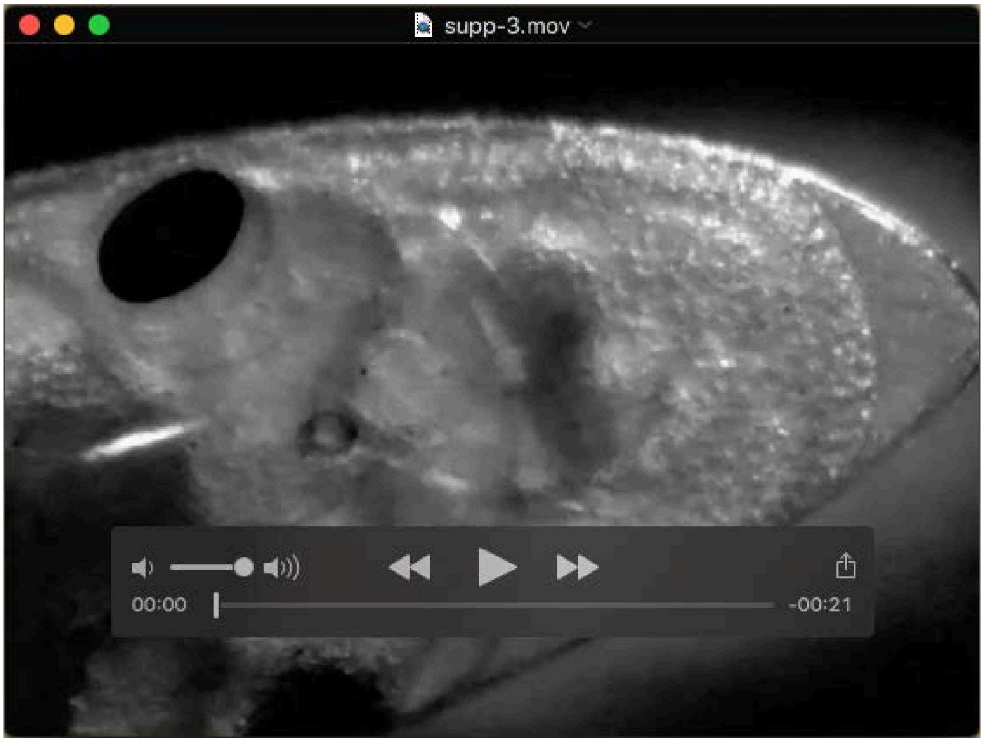

Movie 2. Initial heart beats in *O. insularis*. A Naef stage XV embryo. In this lateral view, two different heartbeats could be detected. We believe these to be the branchial hearts. These hearts do not beat synchronously; the one at the top beats approximately every 2 seconds, while the other beats erratically. There is some movement between the two branchial hearts; however, at this developmental stage is not clear to us if this is the systemic heart.

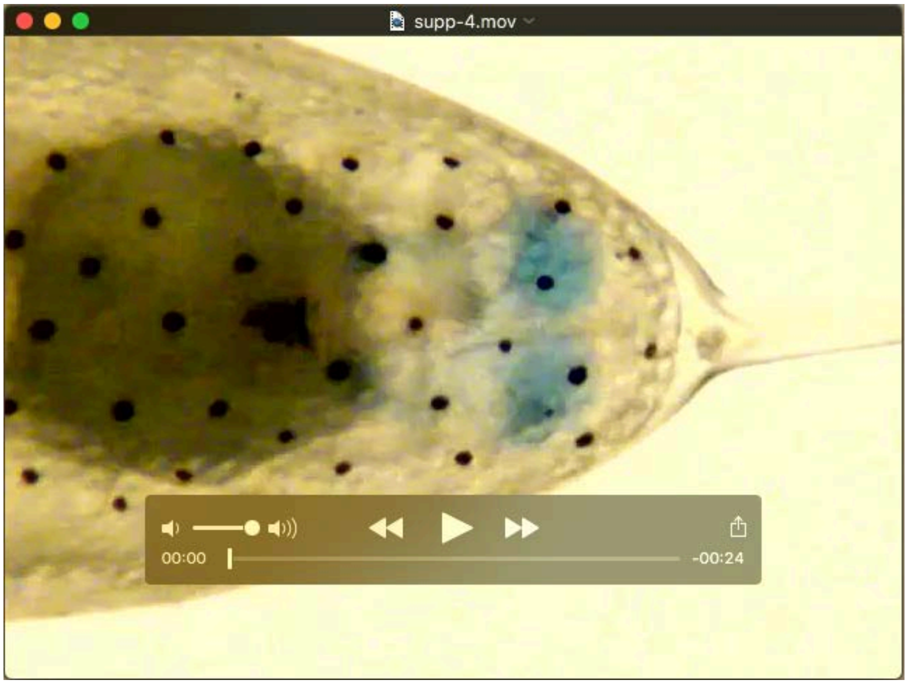

Movie 3. Branchial and systemic hearts from one *O. insularis* embryo. A Naef stage XVIII *O. insularis* embryo shown in a ventral view. For the first 10 seconds of the movie, only the branchial hearts are beating, which can be seen at lateral positions of the body. At this point, the embryo moves, and the systemic heart suddenly starts beating. The systemic heart is located between the two branchial hearts. At this point in development, the three hearts do not seem to beat synchronically.

Biology Open • Supplementary information

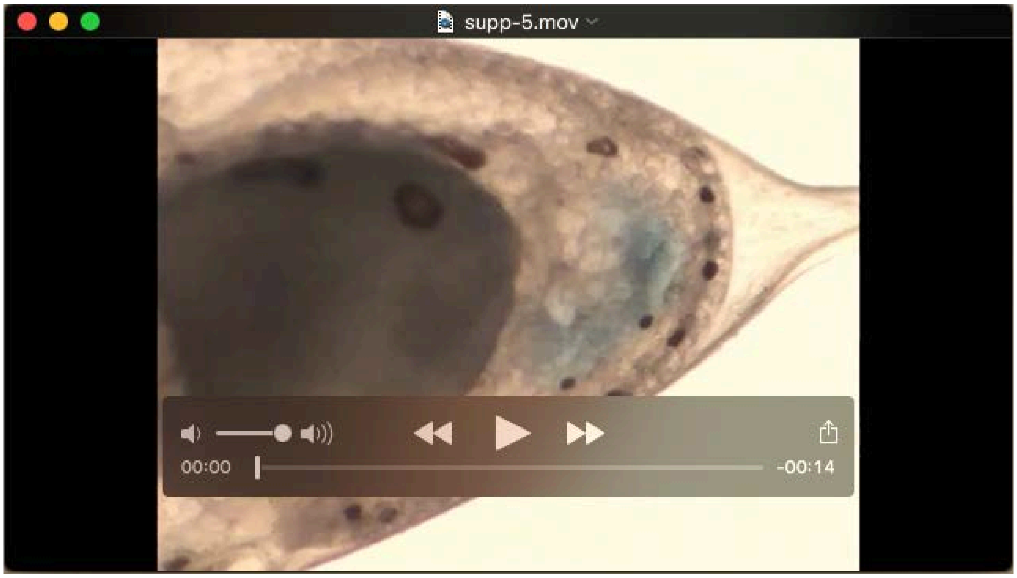

Movie 4. Systemic heart beating in a lateral view from *O. insularis*. Lateral view from a Naef stage XVIII *O. insularis* embryo. From this side, it was easier to follow and analyze the coordination of branchial and systemic heartbeats. These movies are first shown at normal speed and then in slow motion.

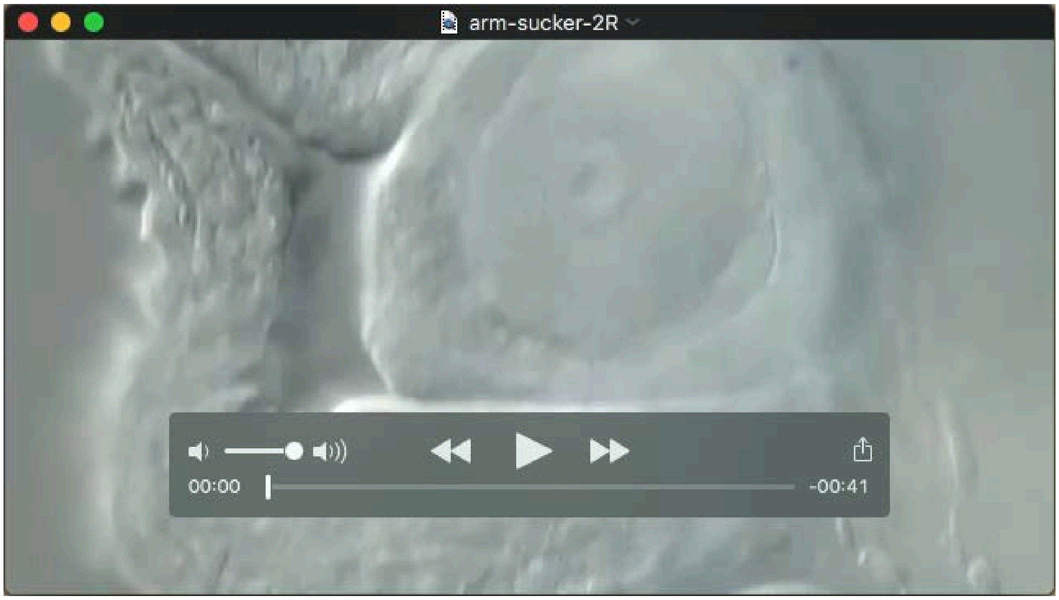

Movie 5. Arms and suckers from an *O. insularis* embryo. This is a Naef stage XVIII embryo of *O. insularis* embedded in agarose and observed using DIC microscopy. Details of one arm and two suckers can be observed at the cellular level; the focal point was switched often to capture details at different depths. During the fast movement of sucker suction, a group of cells surrounding the central part was observed to be drawn towards the central pocket only to return to their original position thereafter. This action is also shown in slow motion. This cellular displacement, clearly governed by muscular contraction, could be related to sucker suction, even during developmental stages.

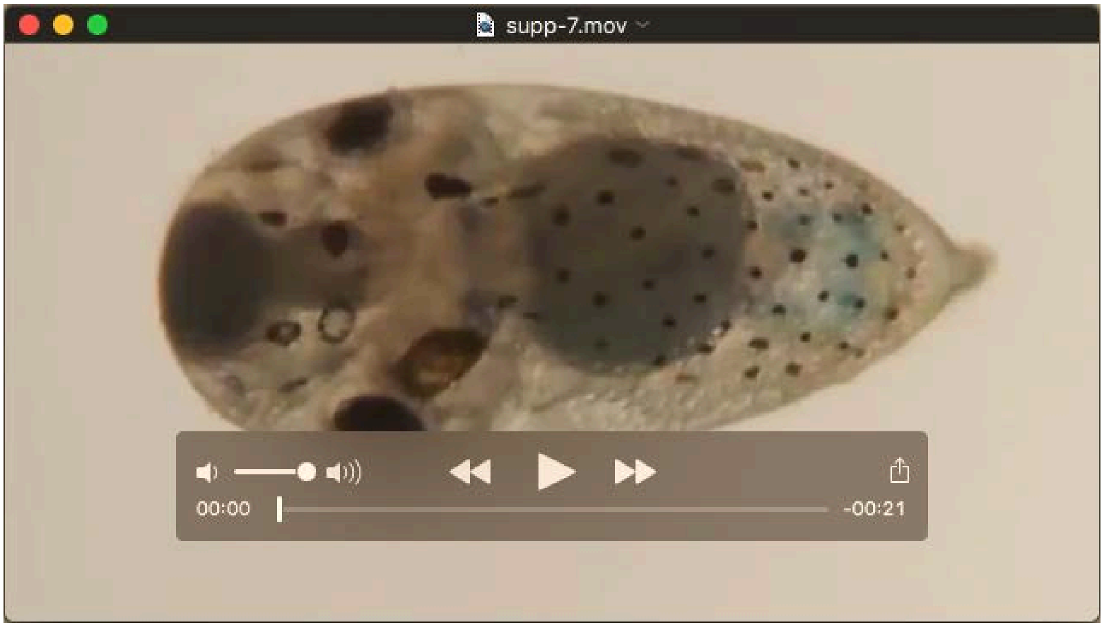

Movie 6. Wave of chromatophore expansion-retraction. A Naef stage XVIII *O. insularis* embryo seen from the ventral side. Sudden spontaneous waves of chromatophores expanding and retracting are initiated at the posterior end and travel towards the anterior side. The wave is spontaneously repeated three times within approximately 7 seconds. At the beginning of the movie, other chromatophores (even at the opposite side) are also expanding and retracting, such as the large chromatophore located by the right eye. At the end of the movie, we show a single expansion-retraction wave in slow motion; when the wave reaches the middle region of the body (over the internal yolk sac), chromatophores at the posterior end are already retracting.

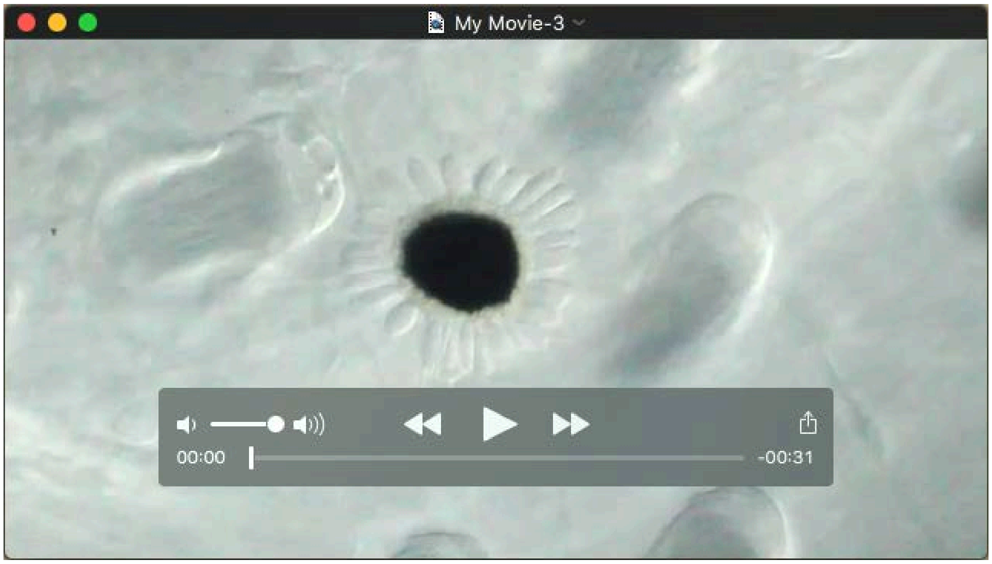

Movie 7. A single chromatophore expansion movement. A Naef stage XX embryo was dechorionated and embedded in a 1% agarose chamber filled with FSW and then placed under an upright microscope with DIC illumination at high amplification (40X). The movie is accelerated four times the normal speed and shows the expansion of a single chromatophore. As a stimulus to induce the movement, we turned on a fluorescence lamp with a rhodamine filter for a few seconds. Muscular fibers alternate in pulling the chromatophore from different directions; consequently, the pigment cell is unevenly stretched at the beginning. Nevertheless, the result is a fully expanded chromatophore by the end of the movie.

Biology Open • Supplementary information
